# The context-dependent role of transforming growth factor-β/miR-378a-3p/connective tissue growth factor in vascular calcification: a translational study

**DOI:** 10.18632/aging.204518

**Published:** 2023-02-13

**Authors:** You-Tien Tsai, Hsiang-Yuan Yeh, Chia-Ter Chao, Jenq-Wen Huang, Chih-Kang Chiang

**Affiliations:** 1 Nephrology Division, Department of Internal Medicine, National Taiwan University Hospital, Taipei, Taiwan; 2 Department of Data Science, Soochow University, Taipei, Taiwan; 3 Nephrology Division, Department of Internal Medicine, National Taiwan University College of Medicine, Taipei, Taiwan; 4 Graduate Institute of Toxicology, National Taiwan University College of Medicine, Taipei, Taiwan; 5 Nephrology Division, Department of Internal Medicine, National Taiwan University Hospital Yunlin Branch, Yunlin County, Taiwan; 6 Department of Integrative Diagnostics and Therapeutics, National Taiwan University Hospital, Taipei, Taiwan

**Keywords:** aortic calcification, connective tissue growth factor, microRNA, transforming growth factor-β, vascular calcification

## Abstract

Background: Vascular calcification (VC) constitutes an important vascular pathology with prognostic importance. The pathogenic role of transforming growth factor-β (TGF-β) in VC remains unclear, with heterogeneous findings that we aimed to evaluate using experimental models and clinical specimens.

Methods: Two approaches, exogenous administration and endogenous expression upon osteogenic media (OM) exposure, were adopted. Aortic smooth muscle cells (ASMCs) were subjected to TGF-β1 alone, OM alone, or both, with calcification severity determined. We evaluated miR-378a-3p and TGF-β1 effectors (connective tissue growth factor; CTGF) at different periods of calcification. Results were validated in an *ex vivo* model and further in sera from older adults without or with severe aortic arch calcification.

Results: TGF-β1 treatment induced a significant dose-responsive increase in ASMC calcification without or with OM at the mature but not early or mid-term VC period. On the other hand, OM alone induced VC accompanied by suppressed TGF-β1 expressions over time; this phenomenon paralleled the declining miR-378a-3p and CTGF expressions since early VC. TGF-β1 treatment led to an upregulation of CTGF since early VC but not miR-378a-3p until mid-term VC, while miR-378a-3p overexpression suppressed CTGF expressions without altering TGF-β1 levels. The OM-induced down-regulation of TGF-β1 and CTGF was also observed in the *ex vivo* models, with compatible results identified from human sera.

Conclusions: We showed that TGF-β1 played a context-dependent role in VC, involving a time-dependent self-regulatory loop of TGF-β1/miR-378a-3p/CTGF signaling. Our findings may assist subsequent studies in devising potential therapeutics against VC.

## INTRODUCTION

Vascular calcification (VC) refers to an adverse vascular pathology characterized by the ectopic deposition of calcium apatite within vascular intima, media and/or rarely adventitia. More than an innocent bystander [1], VC exhibits negative prognostic impact through aggravating survival and increasing cardiovascular risk [2]. Among patients with a higher VC risk, including those of advanced age and with chronic kidney disease (CKD), VC leads to increasing vascular stiffness and an elevated peripheral resistance, rising myocardial afterload and impairing coronary perfusion. The above pathophysiologic combinations thereby increase the risk for heart failure and mortality. Despite the detrimental influences of VC, we are still seeking effective therapies against VC [3]. This gap in VC therapeutics likely results from the incomplete understanding of VC pathogenesis and modulators.

Vascular smooth muscle cells (VSMCs) with phenotypic alterations from a contractile type to a secretory one presumably accounts for the development of medial VC. A plethora of signaling pathways drives and perpetuates this trans-differentiation process, among which reactive oxygen species and pro-inflammatory milieu play an important role [4]. Transforming growth factor β (TGF-β) family members reap much attention amidst VC pathogenesis; one of the TGF-β subfamilies, the bone morphogenetic proteins (BMPs), and their receptors and regulators are well known modifiers for the phenotypic plasticity of vascular wall cells and their progenitors [5]. Another subfamily, the TGF-β, participates heavily in fibrosis promotion and tissue repair responses. However, the relationship between TGF-β subfamily and vasculopathy pathogenesis, including atherosclerosis and VC, remains elusive and is fraught with controversial findings. Experimental data suggest that TGF-β1 suppression predisposed mice to developing atherosclerotic plaques with prominent inflammatory cells [6], while others reported that TGF-β1 contributed to intimal formation of lipid-rich lesions [7]. As for their effect on VC, VSMCs exposed to TGF-β1 showed increased expressions of osteoblastogenesis markers (osteocalcin and alkaline phosphatase) [8]. On the contrary, Shimokado et al. revealed that TGF-β1 treatment attenuated arterial calcification through canonical pathways [9]. These findings cast doubt on the exact influences of TGF-β1 on VC and their clinical implications. Finally, existing studies rarely address the association between circulating TGF-β1 and VC.

In this study, we hypothesized that TGF-β1 actively participated in the pathogenesis of VC, but the influences could be context-dependent. Using *in vitro* and *ex vivo* platforms and clinical specimens for result validation, we aimed to elucidate the experimental and clinical role of TGF-β1 in VC, a dreadful yet hardly treatable condition.

## RESULTS

### Strategy 1: exogenous TGF-β1 effect

**Figure 1:**
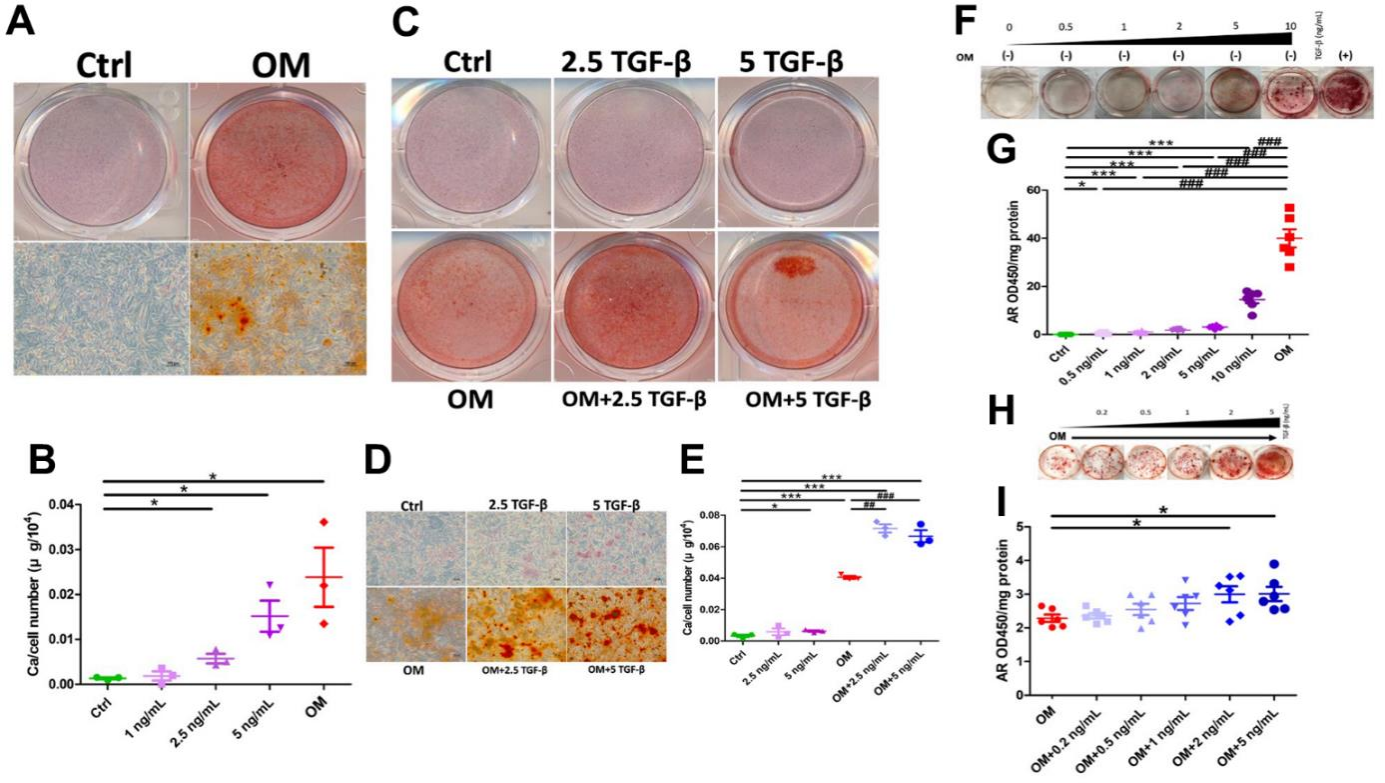
**The influence of exogenously administered TGF-β1 on vascular calcification during the mature phase.** TGF-β1 exhibited a dose-dependent increase in calcification regardless of OM exposure across ASMC of different species. (**A**) Gross (upper row) and microscopic (lower row) view of ASMCs treated with OM, AR-stained (**B**) Calcium quantitation results of ASMCs without or with different TGF-β1 concentrations or OM treatment. (**C**) Gross and (**D**) microscopic view of ASMCs treated without or with TGF-β1 alone, OM alone, or in combination, AR-stained. (**E**) Calcium quantitation results of ASMCs without or with TGF-β1 alone, OM alone, or in combination. (**F**) Human ASMCs without OM and with different concentrations of TGF-β1, stained with AR. Quantitative results are shown in panel (**G**). (**H**) Human ASMCs with OM co-treated with different concentrations of TGF-β1, stained with AR. Quantitative results are shown in panel (**I**). AR, Alizarin red; ASMC, aortic smooth muscle cell; ctrl, control; OM, osteogenic media; TGF-β1, transforming growth factor-β1.

After 7 days of osteogenic media (OM) exposure (the mature phase), treated rat aortic smooth muscle cells (ASMCs) developed prominent Alizarin red (AR) positively stained nodules under gross (Figure 1A, upper row) and microscopic examinations (Figure 1A, lower row), while none was observed in untreated ones. We next incubated ASMCs with increasing concentrations of TGF-β1 and compared calcification propensity to those treated with OM. Results showed that 2.5 and 5 ng/mL TGF-β1 induced 4-fold and 8-fold increased calcification compared to the control group, although the degree of elevation remained lower than that in the OM-treated group (Figure 1B). We subsequently chose 5 ng/mL TGF-β1 in the following experiments. Phenotypically, exogenous TGF-β1 without OM induced mild albeit significant dose-dependent increase in AR positively stained nodules (Figure 1C, 1D, upper row), while TGF-β1 cotreated with OM led to more prominent increase in AR positive nodules compared to that in OM-only and the control groups (Figure 1C, 1D, lower row). OM induced 10-fold greater calcium deposition than the control, while 2.5 and 5 ng/mL TGF-β1 cotreated with OM led to 80% greater calcification severity relative to the OM only group (Figure 1E). We also examined the influences of TGF-β1 on calcification in human ASMCs. TGF-β1 treatment only without OM similarly led to a dose-dependent increase in AR positively stained nodules compared to the control after incubation of 14 days (Figure 1F); this effect took place after TGF-β1 higher than 0.5 ng/mL (Figure 1G). TGF-β1 co-treated with OM also enhanced calcification compared to the OM only group after 7 days of incubation (Figure 1H). We derived that 2 and 5 ng/mL TGF-β1 induced significantly higher calcification severity compared to the OM only group (Figure 1I).

**Figure 2:**
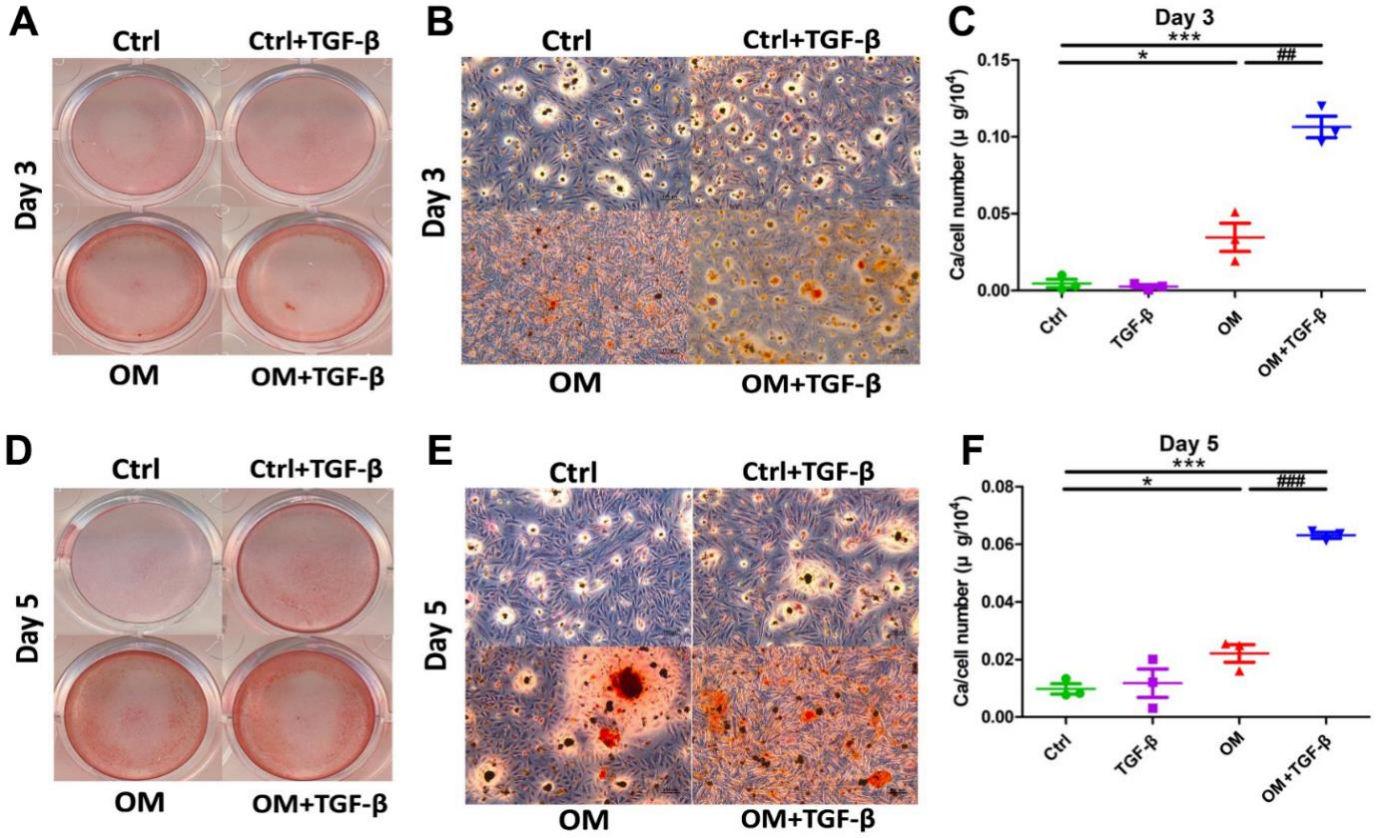
**The context-dependent influences of TGF-β1 on VC, based on different test strategies, during the early and mid- term phases of calcification.** OM and TGF-β1 synergistically increased calcification upon co-treatment. During early (day 3) calcification, ASMCs exposed to TGF-β1 alone, OM alone, or in combination, were stained using AR, with gross (**A**) and microscopic (**B**) images shown. Calcium quantitation results of ASMCs without or with TGF-β1 alone, OM alone, or in combination during early calcification are shown in panel (**C**). During mid-term (day 5) calcification, ASMCs exposed to the same condition were stained with AR (gross, **D**; microscopic, **E**) images shown. Calcium quantitation results during mid-term calcification are shown in panel (**F**). AR, Alizarin red; ASMC, aortic smooth muscle cell; ctrl, control; OM, osteogenic media; TGF-β1, transforming growth factor-β1; VC, vascular calcification.

We next evaluated early (day 3) and mid-term (day 5) influences on calcification posed by TGF-β1. During the early phase of VC, exogenous TGF-β1 without OM did not increase AR positive nodules relative to the control group, while TGF-β1 cotreated with OM led to significantly more AR positive nodules, followed by the OM only group (Figure 2A, 2B). Calcification quantification results also lent support to findings shown on qualitative examinations (Figure 2C). During mid-term calcification, AR positive nodules increased prominently in the OM only and OM cotreated with TGF-β1 groups compared to the control (Figure 2D, 2E), while the TGF-β1 only group exhibited only a trend of increasing calcification compared to the control without significant differences during mid-term calcification (Figure 2F).

### Strategy 2: Endogenous TGF-β1 expressions upon OM exposure

**Figure 3:**
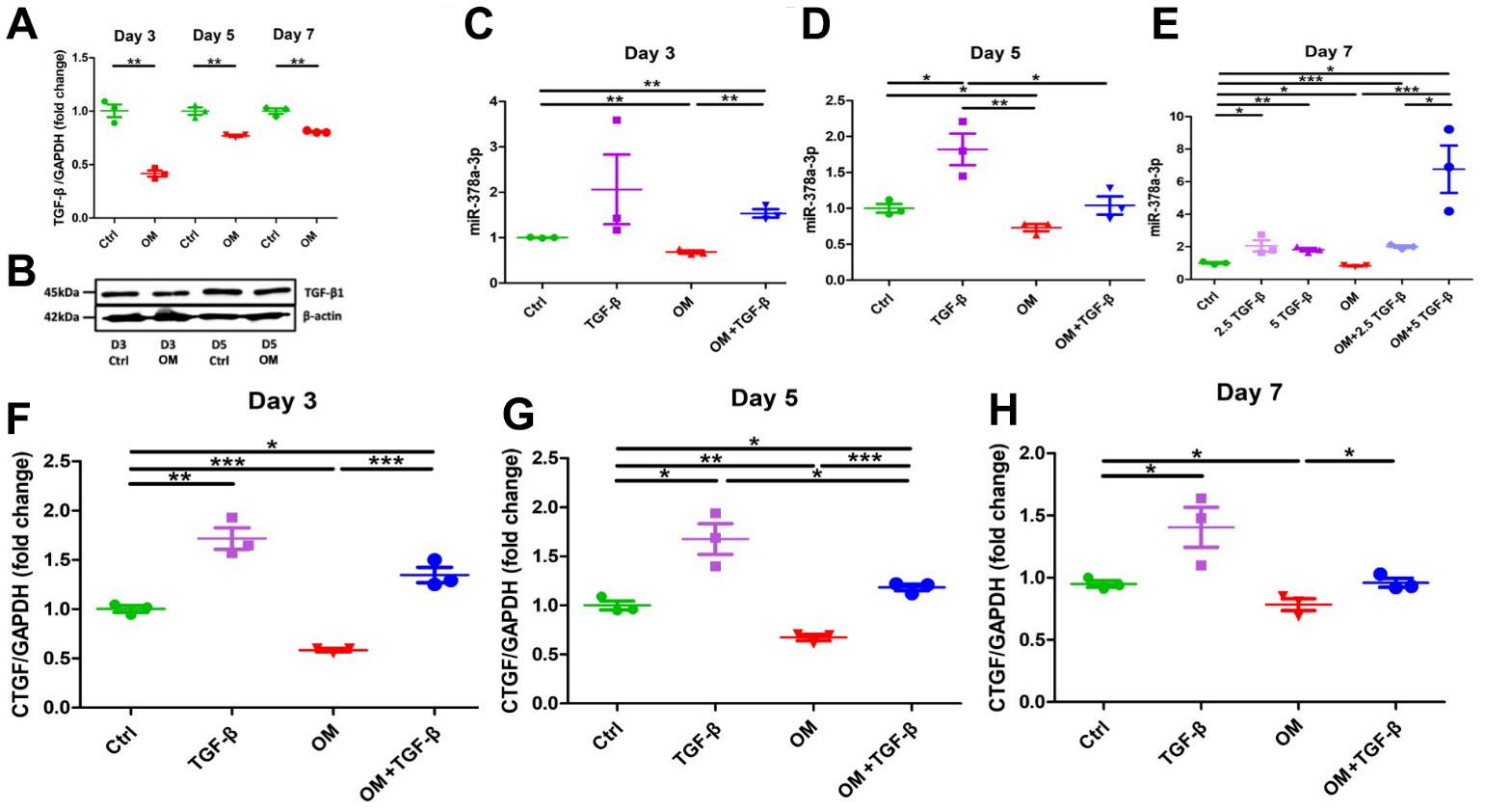
**Changes in the expressions of TGF-β1 and its potential effectors, miR-378a-3p and CTGF upon OM exposure.** TGF-β1 induced an early up-regulation of CTGF, followed by mid-term miR-378a-3p stimulation, while OM suppressed all effectors tested since the early phase of calcification. From early, mid-term to mature VC, OM-induced TGF-β1 (**A**, mRNAs; **B**, western blots), miR-378a-3p (early, **C**; mid-term, **D**; mature, **E**), and CTGF (early, **F**; mid-term, **G**; mature, **H**) expressional changes are shown. ctrl, control; CTGF, connective tissue growth factor; OM, osteogenic media; TGF-β, transforming growth factor-β.

We next examined TGF-β1 expressions in ASMCs treated with OM. TGF-β1 mRNAs were significantly suppressed throughout the early, mid-term, and mature phase of ASMC calcification, with the degree of suppression shrinking toward the mature phase (early, 58%; mid-term, 18%; and mature, 19%) (Figure 3A). TGF-β1 protein levels did not differ between the OM only group and the control during the early phase (OM to control, 1.023) but declined in the OM only group during the mid-term phase (OM to control, 0.785) (Figure 3B). In addition, we examined the canonical receptors of TGF-β1 signaling, Smads in this model (Supplementary Figure 1). Smad1, Smad2, and Smad5 expressions decreased significantly during the mature phase after OM exposure compared to the control, while Smad2 and Smad5 expressions also decreased significantly during the mid-term and early phases, respectively. However, no significant differences were observed regarding Smad3 expression through the entire calcification period.

### Investigating TGF-β1/microRNA/connective tissue growth factor (CTGF) signaling in VC

To verify the biologic consequences of TGF-β1 suppression related to OM exposure, we evaluated the expressions of calcification-regulating miRNA (miR-378a-3p) [10] and connective tissue growth factor (CTGF) levels at different periods of calcification. During the early (Figure 3C), mid-term (Figure 3D), and mature (Figure 3E) phases of calcification, OM consistently and significantly suppressed miR-378a-3p expressions in ASMCs (early, 32%; mid-term 27%; and mature, 17%). Similarly, OM induced significantly lower CTGF expressions in ASMCs over time (early, 42%; mid-term, 33%; and mature, 22%) (Figure 3F–3H, respectively). These findings supported the possibility that miR-378a-3p and CTGF might be downstream effectors of TGF-β1 regarding VC. On the other hand, ASMCs treated with TGF-β1 without OM induced significantly higher miR-378a-3p expression starting from the mid-term (Figure 3D) to the mature phase (Figure 3E) of calcification. The up-regulation of miR-378a-3p induced by TGF-β1 exhibited dose-dependency during the mature phase (Figure 3E). TGF-β1, in the absence of OM exposure, also up-regulated CTGF expressions during the early (Figure 3F), mid-term (Figure 3G), and mature phases (Figure 3H) (early, 71%; mid-term 68%; and mature, 41%). TGF-β1 cotreated with OM led to a milder up-regulation of miR-378a-3p during the early (Figure 3C) and mature phases (Figure 3E) of calcification, and also a milder up-regulation of CTGF during the early (Figure 3F), mid-term (Figure 3G), and mature phases (Figure 3H) of calcification.

**Figure 4:**
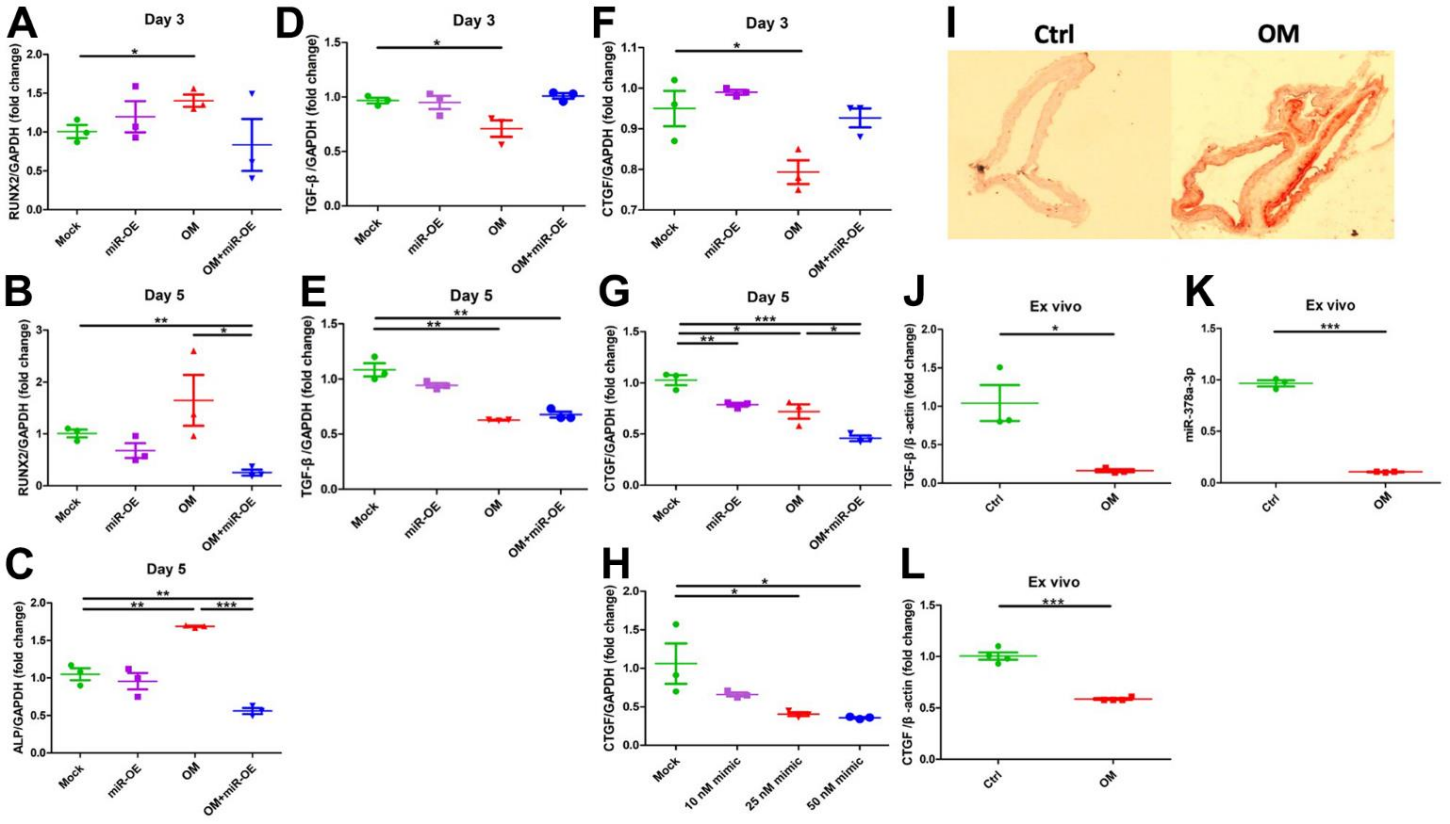
**Functional evaluation of the role of miR-378a-3p during VC and *ex vivo* validation.** Over-expression of miR-378a-3p led to CTGF suppression while remained neutral regarding TGF-β1 expressions. From early to mid-term VC, the expressions of RUNX2 (early, **A**; mid-term, **B**), ALP (**C**), TGF-β1 (early, **D**; mid-term, **E**), and CTGF (early, **F**; mid-term, **G**) in ASMCs without or with miR-378a-3p over-expression or OM treatment are shown. (**H**) The effect on CTGF expression exerted by increasing miR-378a-3p transfection dose. (**I**) AR staining of *ex vivo* aortas without or with OM, with aortic expressions of TGF-β1 (**J**), miR-378a-3p (**K**), and CTGF (**L**) shown. ALP, alkaline phosphatase; AR, Alizarin red; ASMC, aortic smooth muscle cell; ctrl, control; CTGF, connective tissue growth factor; OM, osteogenic media; TGF-β, transforming growth factor-β.

To address whether miR-378a-3p served as an upstream regulator of CTGF, we over-expressed miR-378a-3p in ASMCs and evaluated calcification severity as well as CTGF levels. ASMCs with miR-378a-3p overexpression showed significant down-regulation of RUNX2 (Figure 4A, 4B) during the early and mid-term phases, and also alkaline phosphatase (ALP) (Figure 4C) during the mid-term phase of calcification. These findings indicated that miR-378a-3p overexpression suppressed vascular calcification. miR- 389a-3p overexpression did not alter TGF-β1 mRNA levels during the early (Figure 4D) and mid-term phases (Figure 4E) of calcification. On the contrary, miR-378a-3p overexpression did not alter CTGF expressions during the early phase (Figure 4F) but suppressed CTGF expressions during the mid-term phase (Figure 4G). Importantly, miR-378a-3p overexpression acted synergistically with OM exposure to lower CTGF expressions in ASMCs (Figure 4G). The extent of miR- 378a-3p overexpression paralleled that of CTGF suppression (Figure 4H).

### Result validation in the *ex vivo* model

We further evaluated the expressions of TGF-β1 and miR-378a-3p in an organ culture model. Upon OM incubation, animal aortas exhibited prominent circumferential calcification compared to those without OM (Figure 4I). Calcified aorta exhibited a significant decrease in TGF-β1 (Figure 4J), miR-378a-3p (Figure 4K), and CTGF (Figure 4L) expressions compared to the control.

### Bioinformatic analysis of the TGF-β1/miR-378a-3p/CTGF relationship

**Figure 5:**
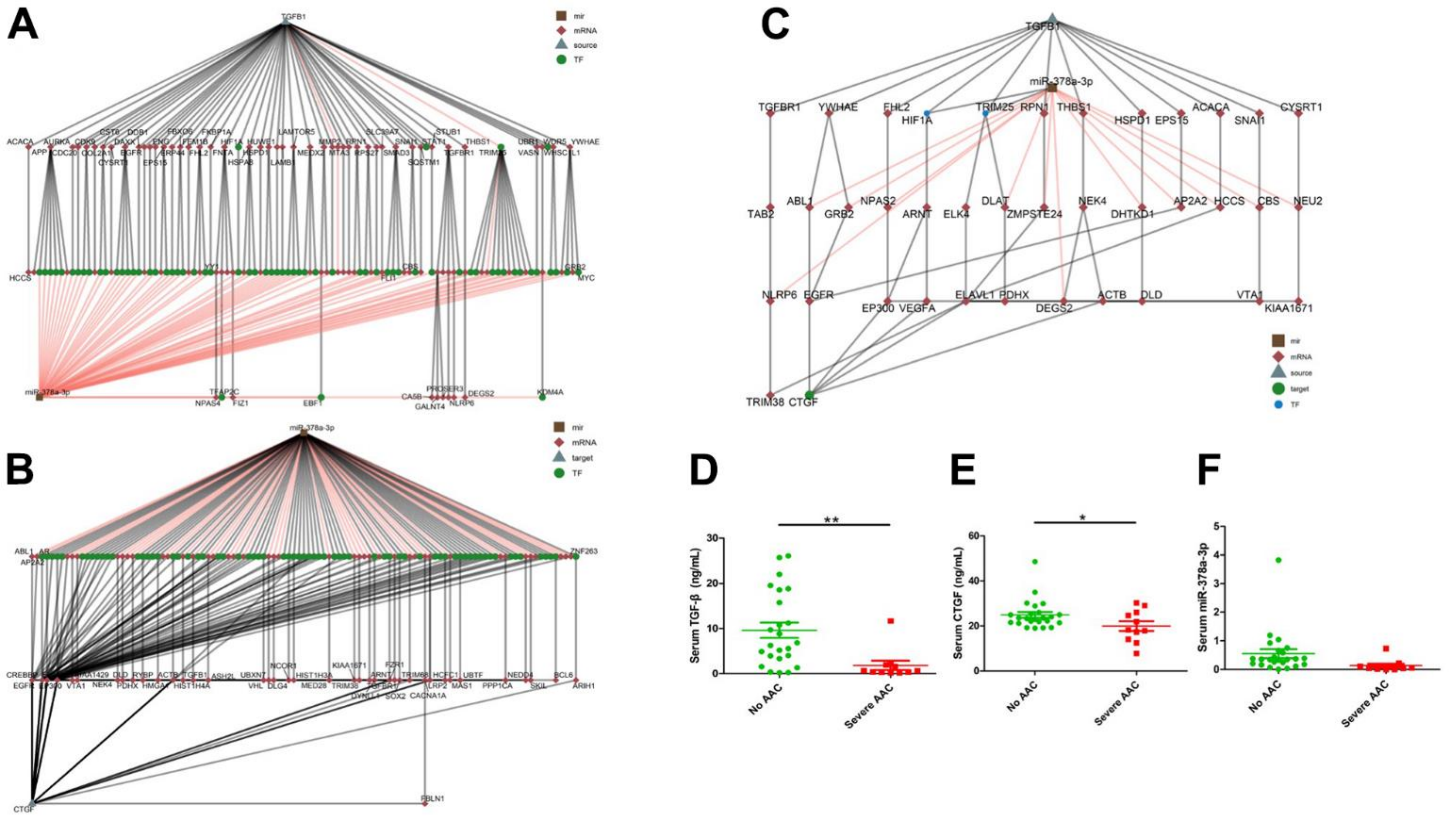
**Bioinformatic analyses of potential links within the TGF-β1/miR-378a-3p/CTGF axis, with multiple mediators uncovered.** The expression levels of TGF-β1/miR-378a-3p/CTGF were found to decrease in sera of patients with severe aortic arch calcification compared to those without. Hierarchical clustering of potential linking nodes/effectors between TGF-β1 and miR-378a-3p (**A**), miR-378a-3p and CTGF (**B**), and between TGF-β1, miR-378a-3p, and CTGF (**C**). Serum TGF-β1 (**D**), CTGF (**E**), and miR-378a-3p (**F**) levels of older adults according to the absence of AAC or presence of severe AAC are shown. AAC, aortic arch calcification; CTGF, connective tissue growth factor; TGF-β, transforming growth factor-β.

The paths between TGF-β1 and miR-378a-3p, and between miR-378a-3p and CTGF were able to form a regulatory network, resulting in a hierarchical search tree space. The node in brown square, red diamond, grey triangle, and green circle denoted the miRNA, mRNA, source/target and transcription factor, respectively. Bioinformatic analyses showed that TGF-β1 modulated the expressions of miR-378a-3p, which potentially influenced the longevity regulating pathway, cellular senescence, and osteoclast differentiation through targeting dedicated gene clusters (Figure 5A). The down regulation of miR-378a-3p further influenced CTGF expressions in stem cells [11], and this connection might go through one to several layers of mediators illustrated in Figure 5B. In addition, CTGF was also a downstream direct target of the TGF-β1 signaling pathway and participated in osteoblastic differentiation involving ASMCs. Although miR-378a-3p expressions were suppressed during VC, leading to the release of CTGF inhibition, CTGF expressions were also decreased following OM-induced TGF-β1 suppression (Figure 5C). Such a negative feedback signal triggered by miR-378a-3p following TGF-β1 signaling points to a potential balance between pro-calcific and anti-calcific influences following OM exposure [12].

### Clinical validation results

We validated our findings in sera from community-dwelling relatively healthy older adults without aortic arch calcification (AAC) and with severe AAC. For circulating miR-378a-3p, we previously showed that miR-378a-3p levels were lower among patients with AAC than those in patients without AAC [13]. In this study, totally 35 participants with a mean age of 75.8 ± 6.0 years and 13 (37.1%) males were identified. Among them, 24 and 11 did not have and had severe AAC, respectively. No significant differences were noted between the two groups regarding their age (*p* = 0.98), body mass index (*p* = 0.26), or the prevalence of morbidities including hypertension (*p* = 0.47) and diabetes mellitus (*p* = 0.94). The AAC group had a significantly lower circulating TGF-β1 (non-AAC vs. AAC, 9.62 ± 8.25 vs. 1.85 ± 3.36 ng/mL, *p* = 0.005) (Figure 5D) and CTGF levels (non-AAC vs. AAC, 24.93 ± 6.39 vs. 19.98 ± 7.11 ng/mL, *p* = 0.048) (Figure 5E) than the non-AAC one. Circulating miR-378a-3p levels were borderline lower in those with severe AAC compared to those without AAC (the former vs. the latter, 0.134 ± 0.207 vs. 0.55 ± 0.763, *p* = 0.086) (Figure 5F).

## DISCUSSION

**Figure 6:**
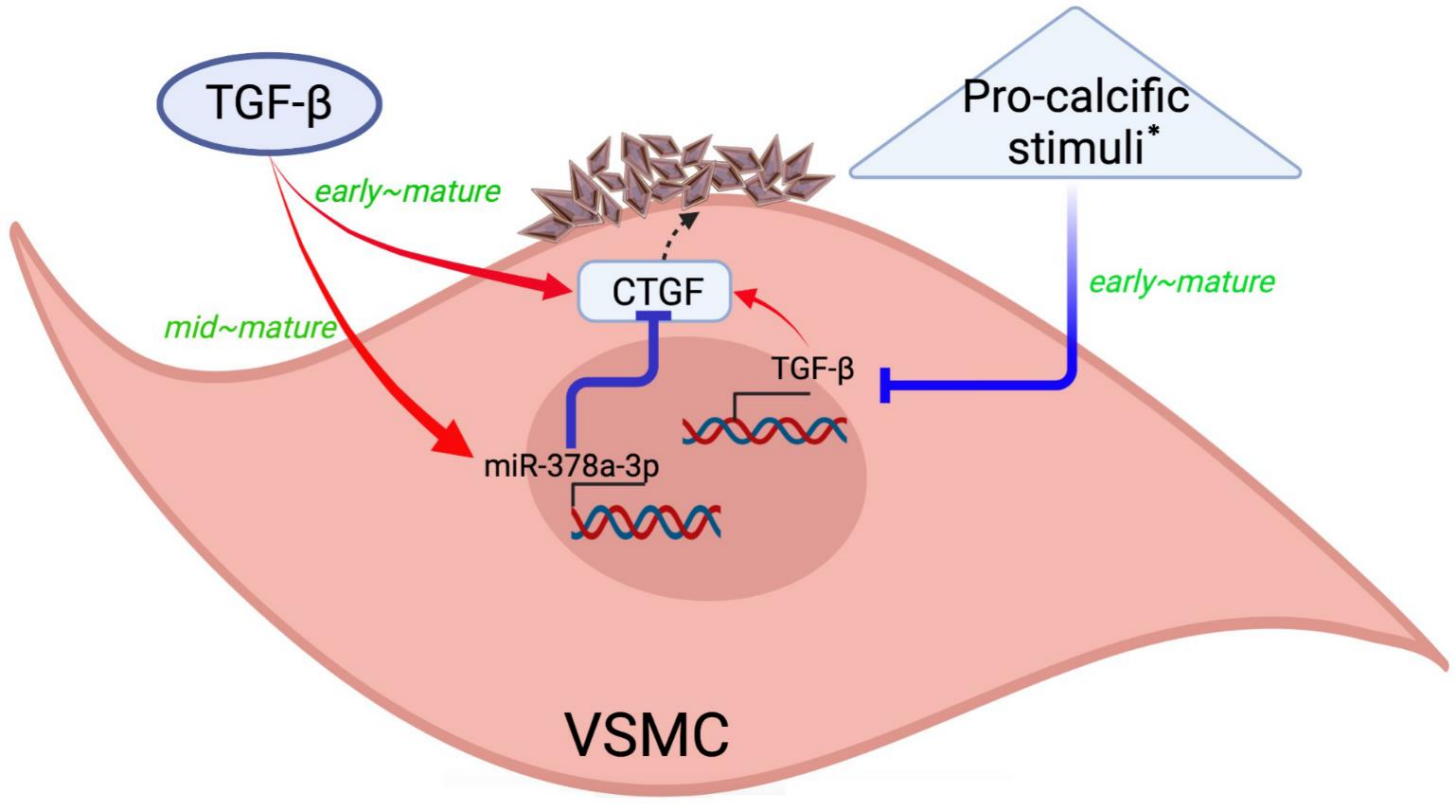
**A graphical illustration of our study findings.** Created with Biorender.com. * Such as high phosphate exposure. CTGF, connective tissue growth factor; TGF-β, transforming growth factor-β; VSMC, vascular smooth muscle cells.

In this translational study, we tested the influence and downstream effectors of TGF-β1 in VC based on the exogenous administration and endogenous expression strategies, and further addressed the temporal changes of relevant molecules. We showed that exogenous TGF-β1 treatment enhanced VC over time, but other pro-calcific stimuli might suppress TGF-β1 expressions during the early, mid-term, and mature phases of calcification, with the degree of suppression fading gradually. TGF-β1 induced miR-378a-3p expression starting from the mid-term phase while augmented CTGF expression earlier. MiR-378a-3p also indirectly suppressed CTGF expressions during VC. TGF-β1 therefore constituted a self-regulatory influence on VC pathogenesis, in which TGF-β1-induced CTGF up-regulation contributed to VC during earlier period of VC, while TGF-β1-induced miR-378a-3p upregulation served a counteracting role during the mid-term to mature phases (Figure 6). On the contrary, the conventional high phosphate-induced VC model might not involve TGF-β1 signaling activation as its core calcification-inducing pathology.

Our findings revealed that TGF-β1 could enhance miR-378a-3p expressions in VSMCs, a regulatory relationship not examined before. An anecdotal study revealed that TGF-β1 up-regulated miR-378a-3p in podocytes through non-canonical pathways including MEK, JNK, and p38 signaling [14], and the disruption of such regulation perturbed glomerular filtration barrier integrity and induced nephrosis. We affirmed that such regulation occurred in VSMCs and potentially participated in the course of calcification. Furthermore, results from Sopel et al. were achieved through screening several hand-picked non-canonical pathways, with positive hits obtained. To establish a more comprehensive view of non-canonical TGF-β1 effectors affecting miR-378a-3p, we cross-linked different database, performed bioinformatic analyses (Figure 5A), and uncovered distinct effectors linking TGF-β1 to miR-378a-3p regulation, including HIF-1α and TRIM25 (Figure 5C). Others reported that the hypoxic environment and associated molecular cascades triggered the up-regulation of miR-378a-3p in osteoblast lineage cells [15], supporting our observations. In addition, we showed that miR-378a-3p served as an upstream regulator of CTGF (Figure 4); similar findings have been reported in other cell types. Hyun and colleagues identified that in hepatic stellate cells, miR-378a-3p overexpression led to CTGF downregulation through directly targeting Gli3 [16]. In another cell type, miR-378a-3p was found to indirectly suppress CTGF expressions [17]. Through integrating multiple data sources, we expanded the putative links between miR-378a-3p and CTGF regulation (Figure 5B). Based on our observations, the TGF-β1/miR-378a-3p/CTGF signaling plays an independent role in VC pathogenesis, although other pro-calcific stimuli may not depend on this machinery.

In this study, we used different periods of observation (early, mid-term, and mature) to better delineate sequential changes in molecular expressions following calcification induction. Although miR-378a-3p was found to regulate CTGF expressions based on our transfection experiments (Figure 4), we also discovered that the timing of elevated CTGF expression preceded that of miR-378a-3p following TGF-β1 treatment (Figure 3). This finding suggested that CTGF acted as an earlier effector for TGF-β1 signaling, while miR-378a-3p was later up-regulated to exert negative feedback. Indeed, others have shown that CTGF was up-regulated as early as 1 day after TGF-β1 exposure [18], while miR-378a-3p expressions were altered following TGF-β1 influenced its effectors 1 day later [14]. The presence of an epigenetic feedback mechanism antagonizing the pro-calcific actions of TGF-β1 partially accounts for the heterogenous findings of TGF-β1 regarding VC in the literature.

It is interesting that TGF-β1 treatment led to an early up-regulation of CTGF, mid-term up-regulation of miR-378a-3p, but did not increase calcification until the mature phase (Figures 2, 3). Several threads of evidence can explain these findings. A previous study showed that the addition of CTGF triggered VSMC calcification only after 6-7 days of exposure [19], and this was mediated indirectly through enhancing the ERK pathway. We previously disclosed that the up-regulation of miR-378a-3p exerted an anti-calcific effect in VSMCs [10]. The combination of the above results thus suggests that exogenous TGF-β1 administration increases CTGF early, but the calcification-promoting effects may take time to onset and potentially be hampered by the mid-term induction of miR-378a-3p expressions. The pro-calcific effect of CTGF then overcomes the feedback effect of miR-378a-3p up-regulation during the mature phase, responsible for the observed increase in calcification at day 7.

We further observed that OM could suppress TGF- β1 expressions in calcified VSMCs. Several pathways might be responsible for this phenomenon. Chang et al. showed that epigenetic regulators, such as miR-26, could increase the propensity for VSMC to calcify through sponging lncRNAs and targeting calcification effectors [20]. Interestingly, miR-26 has been shown to directly target and suppress TGF-β1 expressions, leading to apoptosis induction and autophagy inhibition [21]. Alternatively, vascular calcification might involve angiopoietin/Tie2 axis perturbation [22], while in VSMC, increased Tie2 levels could cause a down-regulation of TGF-β1 expressions [23]. Therefore, our findings are biologically plausible and support the diversity of involved signaling in VC pathogenesis.

CTGF, alternatively known as CCN2, belongs to the CCN family and is an extracellular matrix-associated heparin-binding protein. CTGF plays a critical role in facilitating cellular proliferation, adhesion, migration, and wound repair. Importantly, CTGF has also been shown to assist in atherogenesis. A decade-old study already revealed that significantly increased CTGF expressions could be detected around calcified aortic aneurysms, especially over the vascular media layer [24]. CTGF was subsequently shown to contribute to VSMC calcification through enhancing ERK signaling [19]. Another study further reported that CTGF might serve as the target of miR-26, which antagonized VSMC calcification [25]. More studies are still needed.

During clinical validation, we used OM containing high phosphate to induce cellular calcification, with an aim to recapitulate the VC phenotype in older adults. Older adults carry an exquisitely high burden of VC. Previous studies reported that 16.6% of middle-age to older adults had aortic arch calcification [26], while another discovered that nearly 50% of older adults from a population-based study had incident coronary artery calcification, 2-fold higher than that in younger ones [27]. Risk factors for having VC in older adults include specific gender, greater adiposity or less lean mass, and osteoporosis [28, 29]. Importantly, high serum phosphate has been shown to correlate with a higher VC [30]. High phosphate exposure is frequently used to induce vascular smooth muscle cell calcification *in vitro* to simulate vascular calcification [31]. Therefore, our approach can be a rational one. We further discovered that serum TGF-β1 and CTGF were significantly lower among older adults with VC than those without. There was rare report in the literature addressing the association between serum TGF-β1 and VC. Martin-Gonzalez et al. in a modest group of alcoholic patients, found that aortic arch calcification did not correlate with serum TGF-β1 levels and concluded that TGF-β1 did not constitute a VC risk factor [32]. Several reasons might be responsible for the discrepancy between their findings and ours. Our population is the prototypical risk patients with VC-developing propensity (adults of advanced age), while alcoholic ones are relatively uncommon to have VC. In addition, they revealed an independent relationship between serum TGF-β1 and high-density cholesterol levels [32], suggesting that atherosclerotic calcification might bear a closer relationship with serum TGF-β1 relative to medial calcification. Finally, other sources of serum TGF-β1 might influence such association. Further large-scale studies are needed to validate our findings.

We previously performed a literature review, summarizing the potential pathways involved in OM exposure related VSMC calcification [4]. Plausible pathways include pro-inflammatory cytokines (e.g., tumor necrosis factor-α) and nuclear factor-kB up-regulation, reactive oxygen species induction and anti-oxidative defense attenuation [33], Wnt/β-catenin signaling enhancement, epigenetic dysregulation (e.g., miRNAs other than miR-378a-3p, Sirtuin modulation) [34], and many others. Alterations in any combination of these signaling may contribute to the development of VC upon OM exposure, independent of TGF-β.

Our results have their strengths and limitations. The existing literature poorly characterized the pathogenic role of TGF-β1 in VC with frequent contradictory findings, and we used a more organized approach to disentangle its role in VC. This included the temporal changes of calcification regulatory molecules and miRNAs as well as functional evaluation, thereby unraveling the importance of TGF-β1/miR-378a-3p/CTGF axis in VC, which has not been reported before. However, several limitations remain. The effectors accounting for TGF-β1-induced miR-378a-3p upregulation and miR-378a-3p-mediated CTGF suppression could be multiple, and we uncovered several potential candidates through bioinformatic analyses. These molecules remained to be tested in our future work. We used the high phosphate model to mimic VC *in vitro* and *ex vivo* with validated results derived, but these findings might alter in other VC models outlined in the literature [35]. In addition, there might be other mediators responsible for miR-378a-3p suppression exerted by OM exposure, independent of TGF-β signaling. Prior studies revealed that several long-chain non-coding RNAs might target miR-378a-3p and reduce its expression [36, 37] unrelated to TGF-β. Nonetheless, we did not measure the expressions of these mediators. Nonetheless, we believe that according to our findings, TGF-β1 might not lead to aging-associated VC, although aggravating effect may still exist. More studies are needed to verify our results.

## CONCLUSIONS

In conclusion, we used *in vitro*, *ex vivo* models and clinical specimens to investigate the role of TGF-β1 in VC. TGF-β1 was shown to actively contribute to VSMC calcification when exogenously administered, but upon other pro-calcific stimuli exposure, TGF-β1 and its effectors could be suppressed. We further revealed a self-regulatory loop of TGF-β1/miR-378a-3p/CTGF signaling during VC. Existing studies provide little evidence regarding the linkage between TGF-β and miR-378a-3p, and the effectors of both molecules are relatively unclear in VSMCs. CTGF might serve as one of the potential effectors for calcification promotion, while other plausible intermediates were uncovered through prediction and bioinformatics-assisted screening. Our findings therefore assist in expanding the relevant research landscape.

## MATERIALS AND METHODS

### Experimental utilities

Rat ASMCs were purchased from American Type Culture Collection (ATCC) (A7r5), and maintained in Dulbecco’s Modified Eagle Medium (DMEM) supplemented with 15% fetal calf sera (FCS). Human ASMCs were kind gifts from Dr. Liao and Dr. Shih in Armed Force Taoyuan General Hospital. AR stain and recombinant TGF-β1 were bought from Sigma-Aldrich (St. Louis, MO, USA). Primers for TGF-β, CTGF, RUNX2, ALP, and glyceraldehyde 3-phosphate dehydrogenase (GAPDH) were custom-designed by Mission Biotech. (Taipei, Taiwan), with forward/reverse sequences provided in Table 1. The primer for miR-378a-3p was obtained from QIAGEN (Valencia, CA, USA). Primary rabbit anti-rat TGF-β1 (1:1000) and β-actin (1:10000) were purchased from Abcam (Cambridge, UK) and Sigma, respectively, while secondary goat anti-rabbit antibody (1:3000) were purchased from Abcam (Cambridge, UK).

**Table 1:** Primer sequences used in this study.

**Genes symbol**	**Forward sequence**	**Backward sequence**
*ALP*	**TTGGTCTGGCTCCCATGGTG**	**GCAAAGACCGCCACATCTTCC**
*CTGF*	**CGTAGACGGTAAAGCAATGG**	**AGCAGCAAACACTTCCTC**
*GAPDH*	**AACGGCACAGTCAAGGCTGA**	**ACGCCAGTAGACTCCACGACAT**
*RUNX2*	**ATTTGCCTAACCAGAATGATG**	**TCAATAGGGTCGCCAGACAGAC**
*TGF-β1 (rat)*	**CAACACCTGCACAGCTCC**	**AGTTGGCATGGTAGCCCTTG**
*TGF-β1 (mice)*	**GTGGCTGAACCAAGGAGACG**	**TTGGGGCTGATCCCGTTGAT**
*β-actin (mice)*	**GGTGGGAATGGGTCAGAAGG**	**GGGGTACTTCAGGGTCAGGA**

ALP, alkaline phosphatase; CTGF, connective tissue growth factor; GAPDH, glyceraldehyde-3-phosphate dehydrogenase; TGF-β1, transforming growth factor β1.

### The *in vitro* model of VC and severity quantitation

The detailed procedures of establishing *in vitro* VC have been outlined in our prior work [10, 38]. Briefly, ASMCs were seeded onto culture plates until 80% to 90% confluence, when OM containing high phosphate (mixture of NaH_2_PO_4_ and Na_2_HPO_4_ leading to 2.5 to 3 mM inorganic phosphate concentration) were administered for durations ranging from 3 to 7 days. Such media were exchanged every 2 days. After treatment, ASMCs were washed with phosphate buffered saline and ethanol-fixed. Two approaches were used to measure calcification extent. First, we used AR to qualitatively examine positively stained calcification nodules in the control and OM groups. Second, we used the acid elution method to extract deposited calcium from ASMCs according to our prior work [38]. For rat ASMCs, we used the calcium quantitation kit (Calcium Detection Kit; Abcam, Cambridge, UK) based on cetylpyridinium chloride extraction, using spectrophotometry of OD 560-575 nm absorbance. For human ASMCs, we used an acid extraction method for recovering Alizarin red dye, followed by ammonium hydroxide neutralization and spectrophotometrical detection of calcium amount at OD 450 nm. Calcium quantitative results were normalized to cell number by counting or protein amount measured by a protein amount assay (Biorad, Hercules, CA, USA).

### Reverse transcription-polymerase chain reaction and Western blot

We extracted total RNA from ASMCs and reassured RNA quality using 260/280nm and 260/230nm ratios. This was followed by reverse transcription (RT) and real-time quantitative polymerase chain reaction (qPCR) using the RT kit (Abcam, Cambridge, UK) or miRCURY LNA RT kit (QIAGEN) and SYBR green-based kit (Abcam, Cambridge, UK) or miRCURY LNA SYBR Green PCR kit (QIAGEN), respectively. We calculated fold changes of gene expressions relative to GAPDH or β-actin, using the delta-delta Ct method. The amount of protein of interest was determined using Western blot, with antibodies outlined above.

### Testing the role of TGF-β in VC: time- and dose-dependency

We adopted two strategies for evaluating the role of TGF-β in the process of VC, exogenous administration and stimulated expression upon pro-calcific exposure. During the former experiments, we evaluated the effects of different concentrations (1 – 5 ng/mL) of TGF-β1 on the severity of ASMC without and with OM-induced calcification. During the latter experiments, we evaluated TGF-β1 expression levels in OM-induced ASMC calcification at different temporal periods (early, day 3; mid-term, day 5; and mature, day 7). This arbitrary designation of calcification phases was made based on the pathophysiological observations in other *in vitro* calcification models and calcification-prone animals, which disclosed calciprotein formation in as early as 3 days [39], rapid progression of VC compared to other calcification sites [40], and our prior experimental findings [10, 38]. This was followed by the evaluation of expressions of TGF-β downstream signaling (CTGF) and calcification-regulating miRNA (miR-378a-3p). Existing literature reported that the TGF-β1/CTGF pathway was heavily involved in vascular fibrosis [41], and CTGF up-regulation might contribute to ASMC osteogenic differentiation [19]. We previously showed that miR-378a-3p over-expression suppressed VC progression in ASMCs and animal models [10]. Moreover, TGF-β1 was shown to up-regulate miR-378a-3p expressions in podocytes [42] but whether this action is demonstrable in ASMCs and participates in VC pathogenesis remains unclear.

### *Ex vivo* model of VC

We subsequently tested our *in vitro* findings in an *ex vivo* VC model according to our prior work [10, 38]. The animal experiment protocol, which was part of an integrative VC pathophysiology and therapeutic project, has been approved by the Institutional Animal Care and Use Committee (IACUC) of National Taiwan University College of Medicine (NTU-CM) (NO. 20190048). Briefly, wild type C57BL/6 mice were purchased from LASCO (Taipei, Taiwan), housed and acclimatized in the Laboratory Animal Center of NTU-CM with food and water provided *ad libitum*. Aortas were harvested and subjected to DMEM or osteogenic media, cultured for up to 9 - 14 days. We assessed the expression of TGF-β1 and miR-378a-3p without or with OM and/or exogenously administrated TGF-β1.

### microRNA transfection experiments

To dissect the regulatory relationship between TGF-β, miR-378a-3p, and CTGF, we transfected ASMCs with miR-378a-3p and analyzed the mRNA levels of TGF-β1 and CTGF. Rat ASMCs were seeded onto 24-well plates, and we used Lipofectamine RNAiMAX (Invitrogen) to deliver mature miR-378a-3p (miRCURY LNA miRNA Mimics; QIAGEN). After reassuring transfection efficiency, we performed qPCR for TGF-β1 and CTGF. We also examined whether the degree of miR-378a-3p overexpression influenced CTGF expressions.

### Bioinformatic analysis of the TGF-β/miR-378a-3p/CTGF relationship

We integrated miRNA-gene-transcription factor (TF) networks which considered multiple transcriptional and post-transcriptional interactions from several databases. We also gathered protein-protein interactions from the Biogrid database (https://thebiogrid.org/), containing 4,964 unique proteins and 8,683 interactions involving rattus norvegicus [43], and the TF-miRNA from TransmiR v2.0 database (http://www.cuilab.cn/transmir) which was manually curated from publications [44]. We then extracted targets of the miRNA using the miRNAtap database in R version 3.4 (https://bioconductor.org/packages/release/bioc/html/miRNAtap.html). We predicted targets of miR-378a-3p using all 5 possible target-predicting databases (DIANA, Miranda, PicTar, TargetScan, and miRDB). These tools predicted miRNA targets based on different binding strategies, and we considered candidate targets of miRNAs predicted by at least three tools, and the union of targets was taken as the target output results for a given miRNA. Finally, we adopted the depth-First search (DFS) strategies to identify reachable nodes and paths from source nodes to target nodes during the construction of miRNA-gene-TF networks.

### Clinical validation studies

In this study, we used cryopreserved sera from community-dwelling older adults participating in a cytokine and miRNA study for measuring circulating TGF-β1 and CTGF, based on enzyme-linked immunosorbent assays (ELISAs) (ab100647 and ab261851; Abcam). Their VC severity was determined based on a validated classification scheme for AAC [45], which also demonstrated good applicability to our patients according to our prior work [13, 46]. We identified those without AAC and those with severe AAC, and compared their circulating TGF-β1 and CTGF levels using the Student’s independent *t*-test, in order to validate the clinical implications of TGF-β1 and CTGF in VC.

### Availability of data and material

The raw data for conducting this analysis are will be available upon reasonable request to the corresponding author and approved by relevant authorities.

### Code availability

Custom codes were used in this study.

## Supplementary Material

Supplementary Figure 1
