# Supplementary material for: The context-dependent role of transforming growth factor-β/miR-378a-3p/connective tissue growth factor in vascular calcification: a translational study
Source: Aging (Albany NY). 2023 Feb 13;15(3):830–45. doi: 10.18632/aging.204518 (PMC9970315; doi:10.18632/aging.204518)
Supplement: Supplementary Figure 1 [file aging-15-204518-s001.pdf]

SUPPLEMENTARY FIGURE

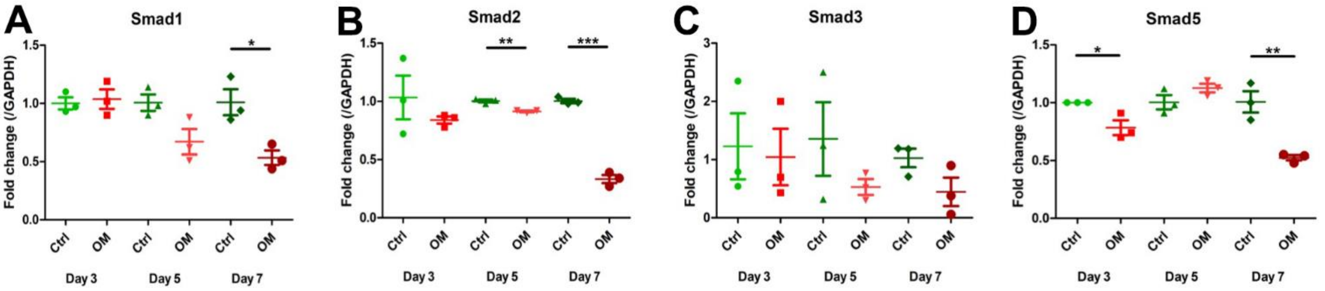

Supplementary Figure 1. Smad (Smad1, **A**; Smad2, **B**; Smad3, **C**; Smad5, **D**) expressions in the OM model. Ctrl, control; OM, osteogenic media.
